# Expansion of droplets during speaking and singing in Japanese

**DOI:** 10.1371/journal.pone.0272122

**Published:** 2022-08-25

**Authors:** Hideaki Kato, Ryuta Okamoto, Sohei Miyoshi, Sho Noguchi, Masakazu Umeda, Yuhei Chiba

**Affiliations:** 1 Infection Prevention and Control Department, Yokohama City University Hospital, Yokohama, Japan; 2 Solution Division, Shin Nippon Air Technologies, Tokyo, Japan; 3 Tokyo Choral Association, Tokyo, Japan; 4 Japan Choral Association, Tokyo, Japan; 5 Department of Psychiatry, Sekiaikai Yokohama Maioka Hospital, Yokohama, Kanagawa, Japan; 6 YUAD, Yokohama, Kanagawa, Japan; Public Library of Science, UNITED KINGDOM

## Abstract

During the COVID-19 pandemic, a number of infection clusters associated with choral singing have been reported. Singing generates droplets and carries the risk of spreading infection. However, no reports have explored droplet flight and aerosol production rates by singing and speaking in Japanese. First, we conducted an observation experiment evaluating the maximum flight distance and number of droplets generated by singing in Japanese, using a high-speed camera and particle counter. Twenty amateur choir members, 10 male and 10 female (five members for each of the four voices), participated in the experiment. Subsequently, although the maximum distance that droplets traveled by singing in Japanese was 61 cm for men (median of 46.5, interquartile range, 36–57) and 56 cm for women (median of 27.5, interquartile range, 20–50), droplets were observed anteriorly and laterally to be up to 66.8 cm. At the singer’s mouth, ≥ 5 μm droplets were observed, whereas not observed at 1 meter toward the front of the singers in women and men, respectively. In German singing, droplets were observed up to 111 cm toward the front of the singer, possibly reflecting differences in pronunciation. In Japanese reading aloud, droplets were also observed up to 47 cm toward the front, whereas no droplet dispersion was observed by speaking the Japanese /a/ vowel or singing with wearing surgical mask toward the front. The aerosols produced when reading singing the /u/ vowels were significantly higher than those in other vowels. When singing in a choral group, keeping a sufficient distance at the front and side is recommended in minimizing infectious spread. If distance is not possible, practicing with /a/ vowels and avoiding consonants may be an alternative method. Our observations lasted only 50 seconds per song, and further observational studies are needed to determine the dynamics of aerosols that stay for long periods.

## Introduction

Choral activity attracted significant attention during the coronavirus disease 2019 (COVID-19) outbreak because of their potential to spread droplets during singing, increasing the risk of infection [1]. Although the risk of spreading droplets varies greatly depending on behavior [2], an increased number of aerosols heightens the risk of severe acute respiratory syndrome coronavirus 2 (SARS-CoV-2) since breathing and speaking also create aerosols [3–5]. Speech aloud and singing raise the respiratory rate and volume resulting in an increased number of produced aerosols compared to when resting [6, 7]. Infection clusters associated with choral activities have been reported from elsewhere in the world. It is reported that 102 of 130 choral participants at a choral concert in the Netherlands [8], 70% of choral rehearsal participants in France [9], and a large cluster in which 53 of the 61 rehearsal participants were confirmed infected in the United States [10]. Cluster infections play a significant role in spreading COVID-19 [4]. Therefore, the prevention of cluster outbreaks is important in continuing choral activities. As observed, half of the people infected with SARS-CoV-2 are still asymptomatic, and can shed the virus two days before symptoms appear [11]. Hence, chorus participants should take care of their physical conditions but also pay attention to avoid exposing droplets.

Conventionally, infective droplets are 5–10 μm in size and have a flying distance of approximately 1 meter [12]. Moreover, in the COVID-19 pandemic, fine size droplets less than 0.5–1 μm in size were generated by speech and singing; the number of produced fine particles is much larger than those of ≥5μm in diameter and played a role in SARS-CoV-2 infection [13, 14]. However, there is no clear boundary between droplets and aerosol, and their size is continuous [15, 16]. Micron-sized small particles, dry soon after speech or singing, and can float for a long time and several meters in the air [17]. Based on a previous study, preventive measures against aerosolized micron-sized particles include choosing a well-ventilated practice venue, like a study conducted in other languages that investigated aerosol dispersion during German singing [18]. Moreover, chorus activities maintaining social distance and considering the distance of droplet flight have also been proposed. However, the dynamics of droplet flight during speaking and singing in Japanese have not yet been reported. Herein, we investigated the droplet flight distance and horizontal dispersion of droplet particles during speaking and singing using a visualization system installed in a clean room to estimate the safety distance for chorus activities.

## Materials and methods

### Experimental settings and participants

On August 23, 2020, the observation study was conducted in a clean room 5.9 m wide, 2.3 m deep, and 2.0 m high and implemented in a building of Shin Nippon Air Technologies, Tokyo, Japan. In this clean room, free particles in the air were eliminated by laminar air flow from the ceiling to the feet for 30 seconds before the observation, after which, the particle-free state was maintained for 60 seconds. This clean room complies with the Class 5 (ISO 14644–1) guidelines. The room temperature was set at 22°C and humidity at 50–60%. Researchers outside the clean room performed visual inspections, showing no apparent floating droplets using a high-speed camera. They also measured the droplet flight distance, and the particle counter sensed no droplets before singing, based on particle counts. Subsequently, the participants were instructed to remain still before observation. At the end of the 30 seconds of air purification, the researcher outside the clean room instructed them to sing or speak. Study participants were recruited on a voluntary basis by the Japan Chorus Association and the Tokyo Chorus Association. All participants were amateurs, consisting of elementary and high school students, adults, and seniors (Table 1). Twenty participants (10 male and 10 female) were selected by voice part: 5 sopranos, 5 altos, 5 tenors, and 5 basses. They were also asked not to eat 30 minutes before the observation. All participants were Japanese and spoke Japanese as their first language. Although droplet flight distance by German singing was compared with Japanese singing, none of the participants were native German speakers, despite their experience in German due to choral singing. The participants provided written informed consent and all confirmed they had no respiratory or febrile symptoms within the past 2 weeks; thus none were excluded from the study. This analysis was approved by the Yokohama City University Hospital Ethical Review Board (2021–008).

**Table 1:** Characteristics of the study participants and the observed maximal distance of droplet flight, and amounts of aerosols produced under 50 s by singing in various ways were shown. All participants were amateurs, comprising elementary and high school students, including adults and seniors, selected by voice parts: 5 sopranos, 5 altos, 5 tenors, and 5 basses.

Participant No.	Age/group	Sex	Voice part	Maximum distance of droplet flight (cm)				Particle counts during 50-second singing					
								At the mouth			1 meter toward the front of the singer		
				Singing in Japanese	Singing in /a/ vowel	Singing in Germany	Reading in Japanese	Particle size in diameter			Particle size in diameter		
								≥ 0.3 μm	≥ 0.5 μm	≥ 5 μm	≥ 0.3 μm	≥ 0.5 μm	≥ 5 μm
1	Elementary school student	Female	Soprano	30				1227	444	33	46	19	0
2	Elementary school student	Female	Alto	13				751	475	38	51	15	0
3	High school student	Female	Soprano	56				1319	649	27	58	10	0
4	High school student	Female	Alto	22				663	481	38	19	7	0
5	High school student	Male	Tenor	57				961	402	2	25	8	0
6	High school student	Male	Bass	19				134	81	2	11	2	0
7	University student	Male	Tenor	58				129	73	1	6	2	0
8	University student	Male	Bass	51				94	61	1	0	0	0
9	Adult (group 1)	Female	Soprano	54				2949	1707	9	0	0	0
10	Adult (group 1)	Female	Alto	36				1021	548	17	20	10	0
11	Adult (group 1)	Male	Tenor	61				730	337	7	9	2	0
12	Adult (group 1)	Male	Bass	46				1339	711	1	5	1	0
13	Adult (group 2)	Female	Soprano	25	0	33	28	661	272	4	25	3	0
14	Adult (group 2)	Female	Alto	23	0	58	20	568	302	2	13	3	0
15	Adult (group 2)	Male	Tenor	42	0	111	44	771	387	1	8	6	0
16	Adult (group 2)	Male	Bass	44	0	93	47	7	3	1	5	2	0
17	Aged	Female	Soprano	49				1551	698	24	27	7	0
18	Aged	Female	Alto	0				1776	892	9	21	5	0
19	Aged	Male	Tenor	47				2010	985	12	7	2	0
20	Aged	Male	Bass	8				2305	1202	3	14	5	0

### Measurement of flight distance of droplet during singing in Japanese

**Fig 1:**
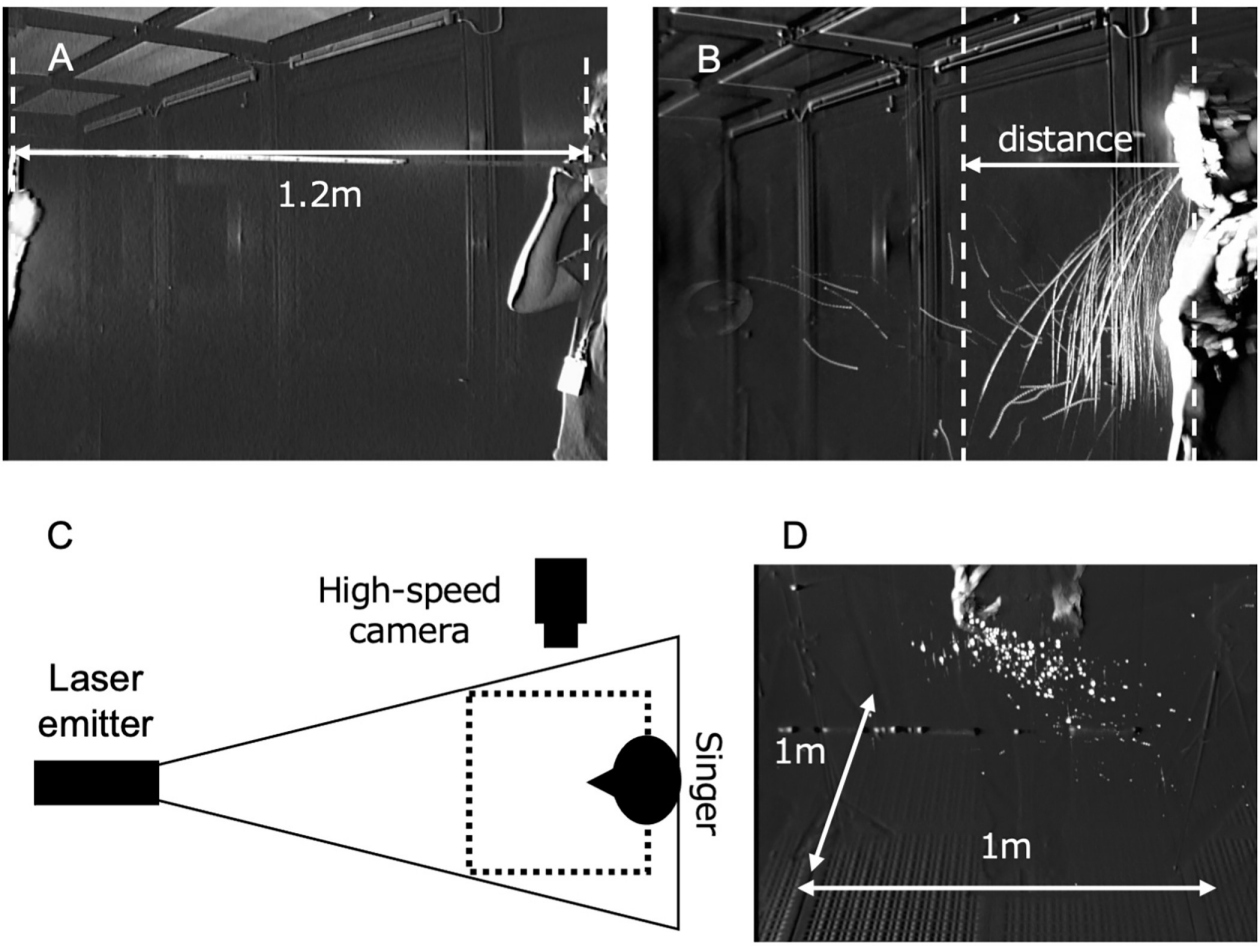
Schematic of the measurement of droplet flight distance and expansion of horizontal plane. Droplet particle flight distance was measured using a laser, a light emitting diode (LED) sheet, and a high-speed camera (A, B). The camera angle was set at a distance of 1.2 m on the screen, and the droplet flight distance was calculated proportionally from 1.2 m. Visualization of the horizontal direction of the droplet particles generated during singing in Japanese (C, D). A laser on a sheet was irradiated at a height of 1 m from the ground and visualized from diagonally upward.

A light emitting diode (LED) sheet (Parallel Eye D, Shin Nihon Air Technologies, Tokyo, Japan) and an ultra-sensitive camera (Eye Scope, Shin Nihon Air Technologies) were used to measure the distance of droplet particles. The Parallel Eye D and the Eye Scope camera are capable of visualizing particles ≥ 5 μm, as previously reported [19]. The subjects wore goggles for eye protection and were exposed to LED sheets at the front. An ultra-sensitive camera was placed at 90 degrees from the study participants to observe droplet particles from the side. The camera angle was set at 1.2 m, and multiple exposures of the trajectory of the droplet particles for 50 s length were taken. The farthest horizontal distance of observed droplet at the subjects’ waist level was measured. The observation period was 50 s because droplets were observed on floors and other surfaces, except for areas around the singers. Moreover, the cleanliness of the clean room could not be guaranteed. We also omitted some fine particles that deviated from the trajectory observed during the observation period by distance measurements, which were considered fine aerosols that dried during the flight. Therefore, this study measured the flying distance of droplets flying in trajectory patterns. We chose the Japanese song “Ode to the Earth (Daichi-San-Sho)” (lyrics: Atsuo Ohki; music: Makoto Sato) because it is widely sung in Japanese schools, and participants sang it from the 43rd measure to the end of the 48-measure song (average 45 seconds). The relevant parts included piano to fortissimo dynamics in strong and weak symbols. The farthest distance was used as the flight distance of the droplet particles (Fig 1A and 1B).

### Measurement of droplet expansion of horizontal plane during singing in Japanese

To evaluate the horizontal spread of droplet particles, a 532 nm green laser (Parallel Eye H, Shin Nihon Air Technologies) sheet was irradiated horizontally in front of the participants at a height of 1 m above the floor. A high-speed camera was set up at 2 m above the floor, and the spread of droplets on the horizontal plane was measured by correcting with a distance marker set up beforehand (Fig 1C and 1D). Droplets dispersed during singing were flashed continuously by Parallel Eye D. Scattering light from droplets was then recorded and multiple exposures for 50 seconds length with the Eye Scope. The mouth of the singer was made to be the zero of the coordinates, and the luminous points of the droplets recorded on the horizontal plane by the camera installed at the top were plotted on the X-Y plane.

### Measurement of the number of produced aerosols during singing

The number of aerosol by singing and speaking was measured using a particle counter (AeroTrak 9303, Tokyo Dylec Corporation, Tokyo, Japan). The particle counter was designed to sample air at 2.83 m^3^/min and count droplet particles with diameters ≥ 0.3 μm, ≥ 0.5 μm, and ≥ 5 μm. The particle counters were adjusted in height and placed on the same horizontal plane so that the inhalation area faced the singer’s mouth. Particle counters were placed 10 cm and 1 m in front of the mouth. Since droplets <1μm evaporate in 0.001 s and lose their initial velocity in 1/10^6^ s, is assumed that the aerosolized droplets and the number of aerosols inhaled directly from the singer into the particle counter were observed [20]. Measurements was taken every 0.2 s, and the total amount at 50 s after the start of singing were counted (S1 Fig).

### Comparison of flight distance of droplets between singing in German and singing the Japanese A vowel

As comparison for singing in Japanese, the subjects were asked to sing the rehearsal mark M of the fourth movement of Symphony No. 9 “An die Freude” (composed by Ludwig van Beethoven) in German. In addition, the subjects were instructed to sing the song with the /a/ vowel in Japanese while wearing a surgical mask, and measured the distance of the droplets.

### Measurement of droplets by vowels and consonants in Japanese vocalization

The Japanese syllabary consists of five vowels (/a/, /i/, /u/, /e/, and /o/), the flat consonants (“a,” “k,” “s,” “t,” “n,” “h,” “m,” “y,” “r,” and “w”), and voiced consonants (“g,” “z,” “d,” “b,” and “p”). For example, the K column includes KA, KI, KU, KE, and KO. The exceptions are WI and WE in the W column, which are not included in the Japanese syllabary. To evaluate the differences in the amount of droplets produced depending on the pronunciation, a Type-S (Shin Nihon Air Technologies Co., Ltd.), which visualize the droplet particles, was placed 10 cm in front of the subject’s mouth, and the number of particles ≥ 0.5 μm passing through an area of 20 × 5 cm was counted every 1/30 second. At the same time, video recording was performed, and the number of particles passing through each sound wave was counted. Since the type-S was installed horizontally and placed near the mouth, the observation was made continuously after air purification until the Japanese syllabary for approximately 100 seconds was pronounced. Moreover, a sound pressure meter and recorder were set up to check the consistency of each pronunciation (S3 Fig).

### Statistical analysis

Continuous data, comprising distances comparing gender differences, including droplet counts observed by mouth comparisons, 1 m ahead of singers, are presented as mean and 95% confidence interval (CI) or median and interquartile range (IQR, Q1–Q3). Categorical data are presented as numbers and percentages. Data were analyzed using the two-tailed Mann–Whitney U-test to compare continuous variables between two groups for non-paired data, and the Wilcoxon matched-pairs signed-rank test for paired data. Multivariate regression analysis was performed to compare the amounts of droplets produced per vocalization. Statistical analyses were performed with Prism 9 (GraphPad Software, San Diego, CA, USA). *P*-values < 0.05 were considered statistically significant.

## Results

### Flight distance of droplets forward during singing in Japanese

**Fig 2:**
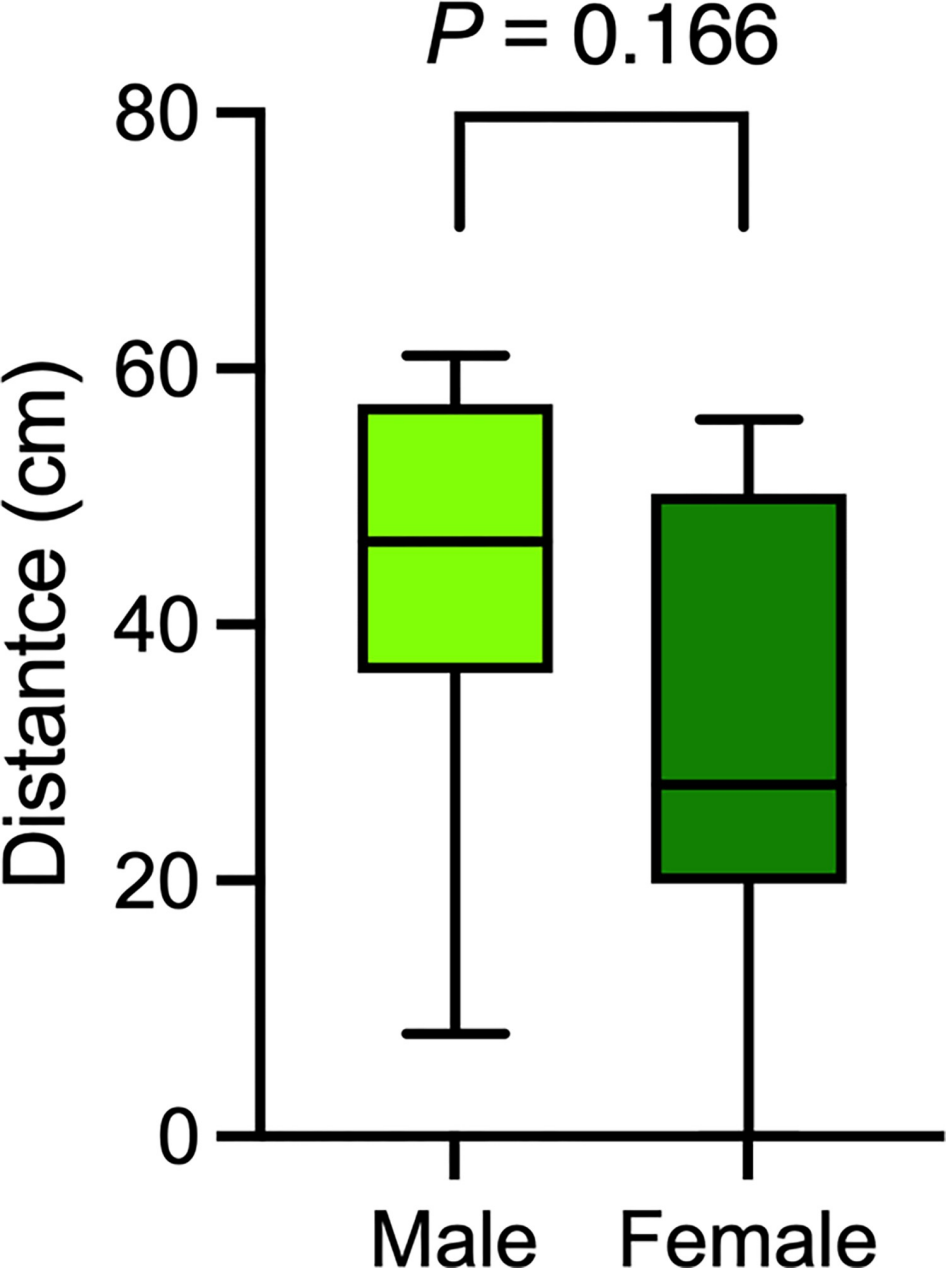
Measurement of droplet dispersal distance when singing in Japanese. Droplet distance of Japanese singing by ten males and ten females. No significant difference was found between the male and female singers.

**Fig 3:**
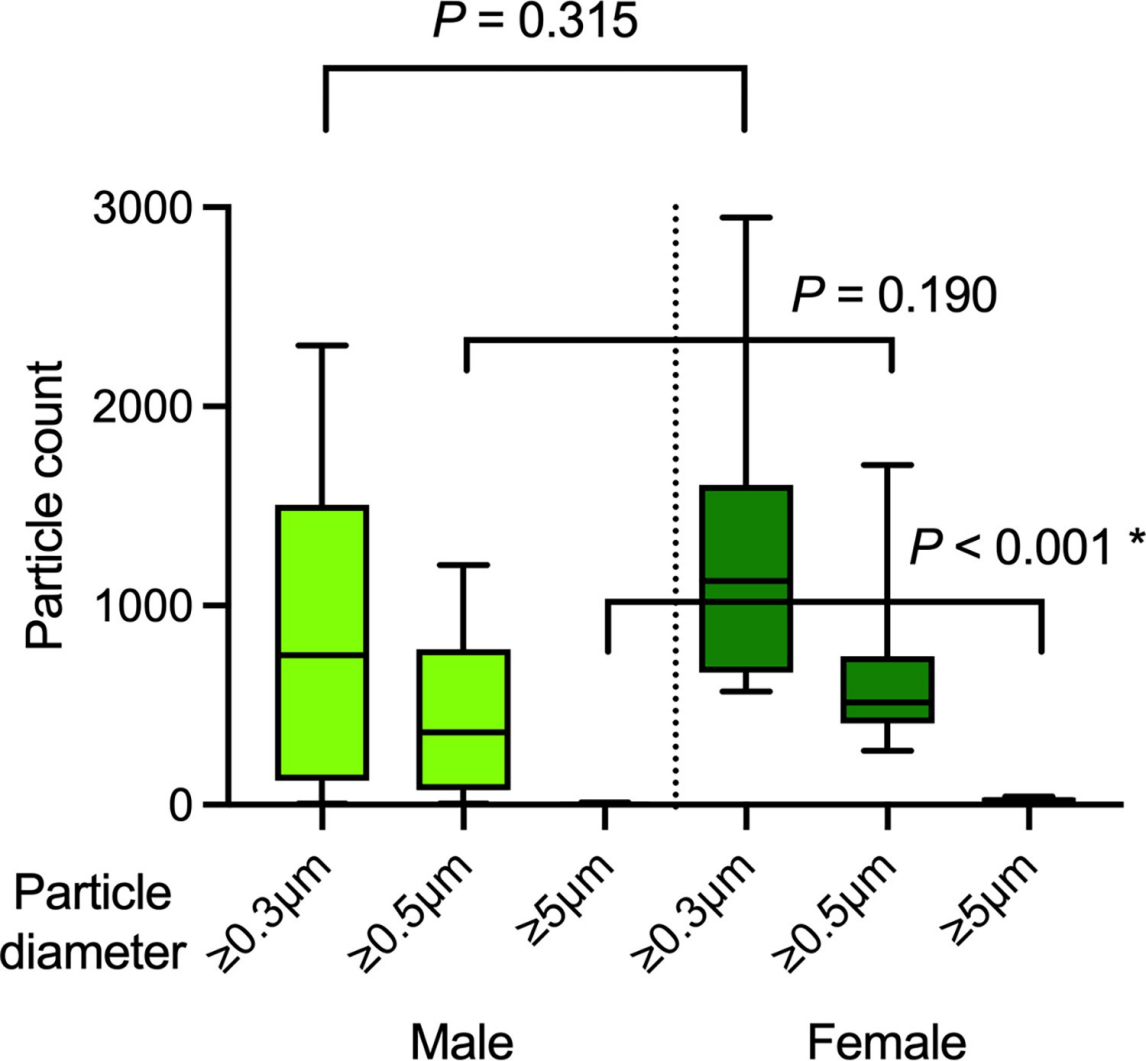
Number of aerosol per particle size at the mouth (A) and 1 m in front of the mouth (B) when singing in Japanese. The number of particles ≥ 5 μm in size was higher in the female participants at the mouth, and particles ≥ 0.3 μm and ≥ 0.5 μm in size were higher in the female at 1 m toward the front. *Significant difference.

The flight distance of droplets and the number of particiles produced in Japanese singing was observed in 10 male and 10 female singers (Table 1). The median droplet flight distance was 43 cm (IQR 22.3–53.3) for all 20 participants. Comparing male and female participants, the maximum flight distance was 61 cm (IQR 36.0–57; median, 46.5 cm) for the male participants and 56 cm (IQR 20–50; median, 27.5 cm) for the female ones (*P* = 0.166), no significant differences were found (Fig 2). The maximum flight distance per voice group (5 singers each) was 56 cm (median 49 cm, IQR 27.5–55) for soprano, 36 cm (median 22 cm, IQR 6.5–29.5) for alto, 61 cm (median 57 cm, IQR 44.5–59.5) for tenor, and 51 cm (median 44 cm, IQR 13.5–48.5) for bass. The median number of aerosol particles at the mouth generated by singing and analyzed by sex was 750.5 (IQR 120.3–1507) for males vs 1124 (662.5–1607) for females (*P* = 0. 315) for ≥ 0.3-μm particles in diameter; 362 (70.0–779.5) for males vs. 514.5 (408.5–746.5) for female (*P* = 0.190) for ≥ 0.5-μm particles; and 1.5 (1.0–4.0) for males vs. 20.5 (7.8–34.3) for females (*P* < 0.001) for ≥ 5-μm particles (Fig 3). The median number of aerosol particles ≥0.3 μm and ≥0.5 μm in diameter dispersed at 1 m toward the front was 7.5 (5.0–11.8) for males vs. 23.0 (17.5–47.3) for females (*P* = 0.006) and 2.0 (1.75–5.75) for males vs. 7.0 (3.0–11.25) for females (*P* = 0.020), respectively (Fig 3). No particles ≥ 5 μm in size were observed at 1 m toward the front.

### Horizontal expansion of droplets produced when singing in Japanese

**Fig 4:**
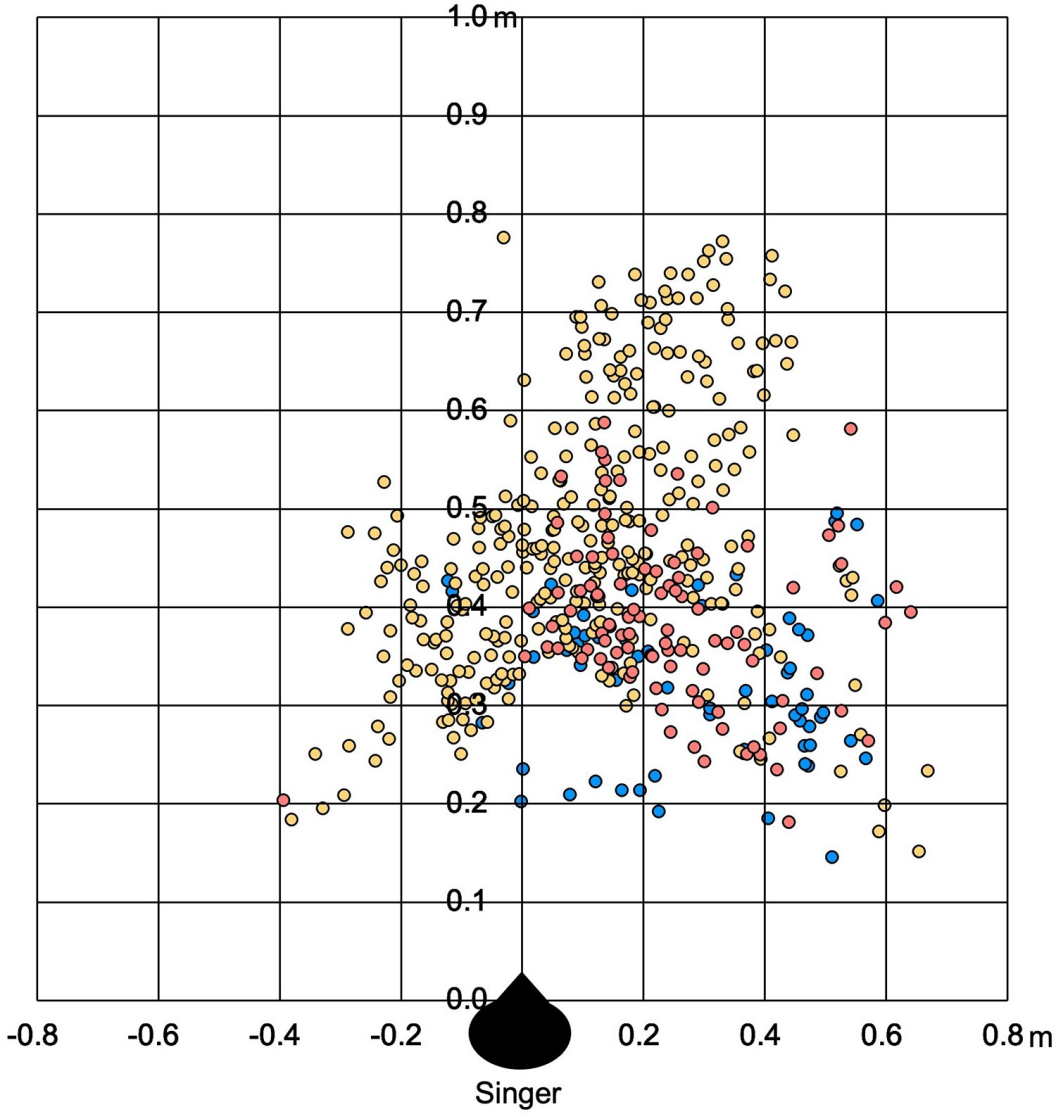
Horizontal expansion of droplets produced when singing in Japanese. The droplets plotted were for soprano (blue), bass (red), and tenor (yellow) singers. Each singer’s droplet, observed during 40 s of singing, were photographed using multiple exposures, with the farthest observed point being the longest distance of droplet flight. The circles are projections on a plane of the droplets observed during each singer’s 50-s singing period. Moreover, maximum horizontal expansions were observed at 77.6 cm toward the front and 66.8 cm to the right.

We evaluated the horizontal spread of droplets using three study participants (soprano, tenor, and bass) belonging to an adult choral group who were selected to sing in Japanese, as it was considered that the adult choral group participants reflected the most representative results of the choral population. Alto did not sing due to a prevented singer’s fatigue. Furthermore, each singer’s droplets observed during 40 s of singing were subsequently photographed using multiple exposures, with the farthest observed point being the longest distance of droplet flight. The droplets produced by the three singers were plotted in an overlapping fashion. The male tenor singer (yellow) showed the greatest dispersion of droplets, with droplet particles extending 77.6 cm toward the front, 66.8 cm to the right, and 38.0 cm to the left (Fig 4). The other two singers (soprano and bass) produced droplet distances of 58.7 cm and 49.6 cm toward the front, 64.1 cm and 58.7 cm to the right, and 39.4 cm and 12.2 cm to the left, respectively. All three were right-handed.

### Comparison with singing in German, reading aloud, and vocalizing

**Fig 5:**
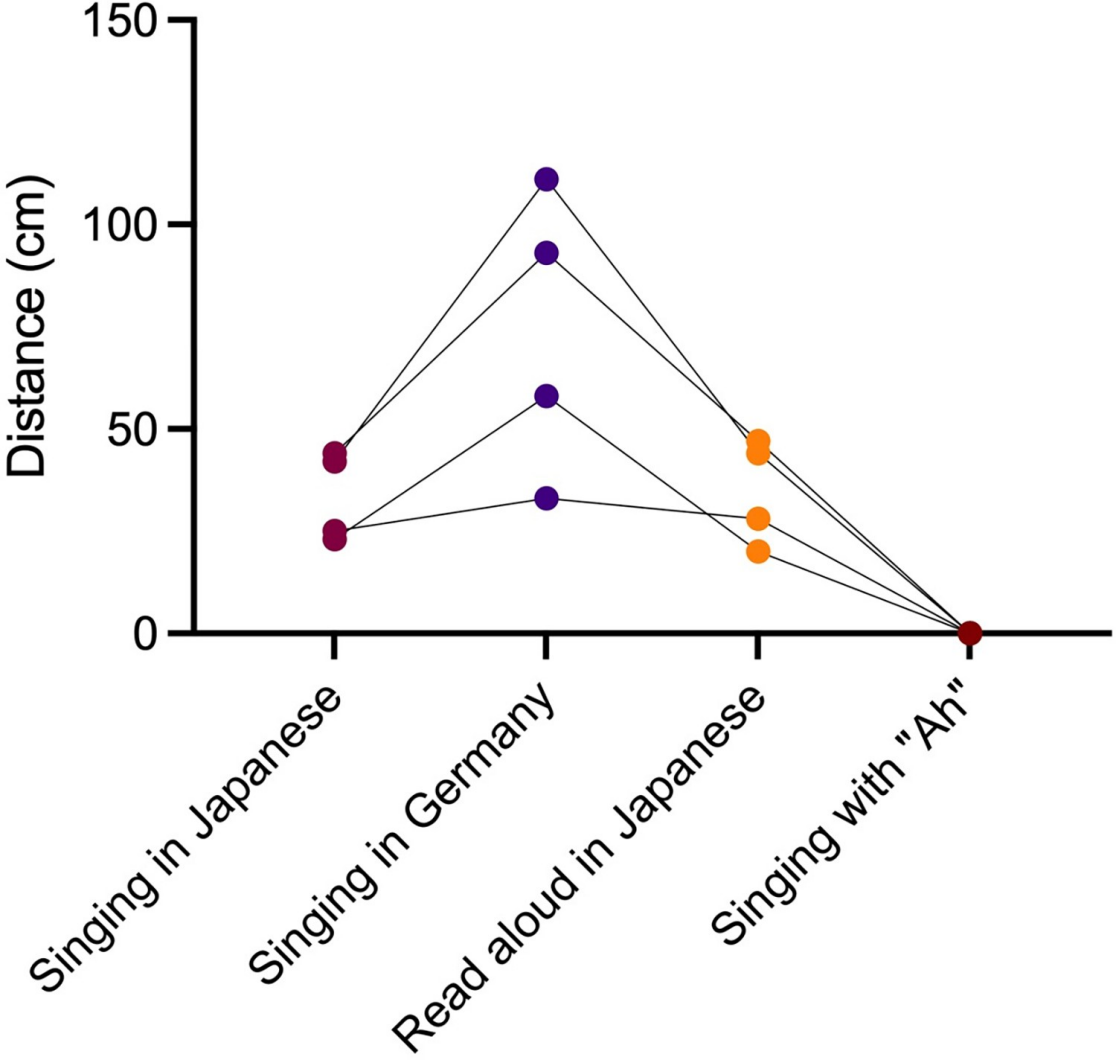
Droplet flight distances produced singing in Japanese and German, reading aloud in Japanese, and singing the A vowel in Japanese. No significant difference was found between reading aloud and singing in Japanese, and no droplet production was observed when singing the A vowel.

To examine the differences in the ways of droplet production, we compared the droplet distances produced by singing in German, reading aloud in Japanese, and singing the A vowel in Japanese (Fig 5). A total of four participants from each part of the adult chorus group participated in this observation. The maximum distance of the droplet particles was 44 cm (median 33.5 cm) for singing in Japanese, 111 cm (median 75.5 cm) for singing in German (*P* = 0.125 compared with singing in Japanese), and 47 cm (median 36 cm) for reading aloud in Japanese (*P* = 0.625 compared with singing in Japanese). However, no droplet production was observed when singing the A vowel in Japanese. In addition, when the participants in this study were asked to sing in German while wearing surgical masks, no droplets were seen in the visualization (S3 Fig).

### Vocalization of the Japanese syllabary

**Fig 6:**
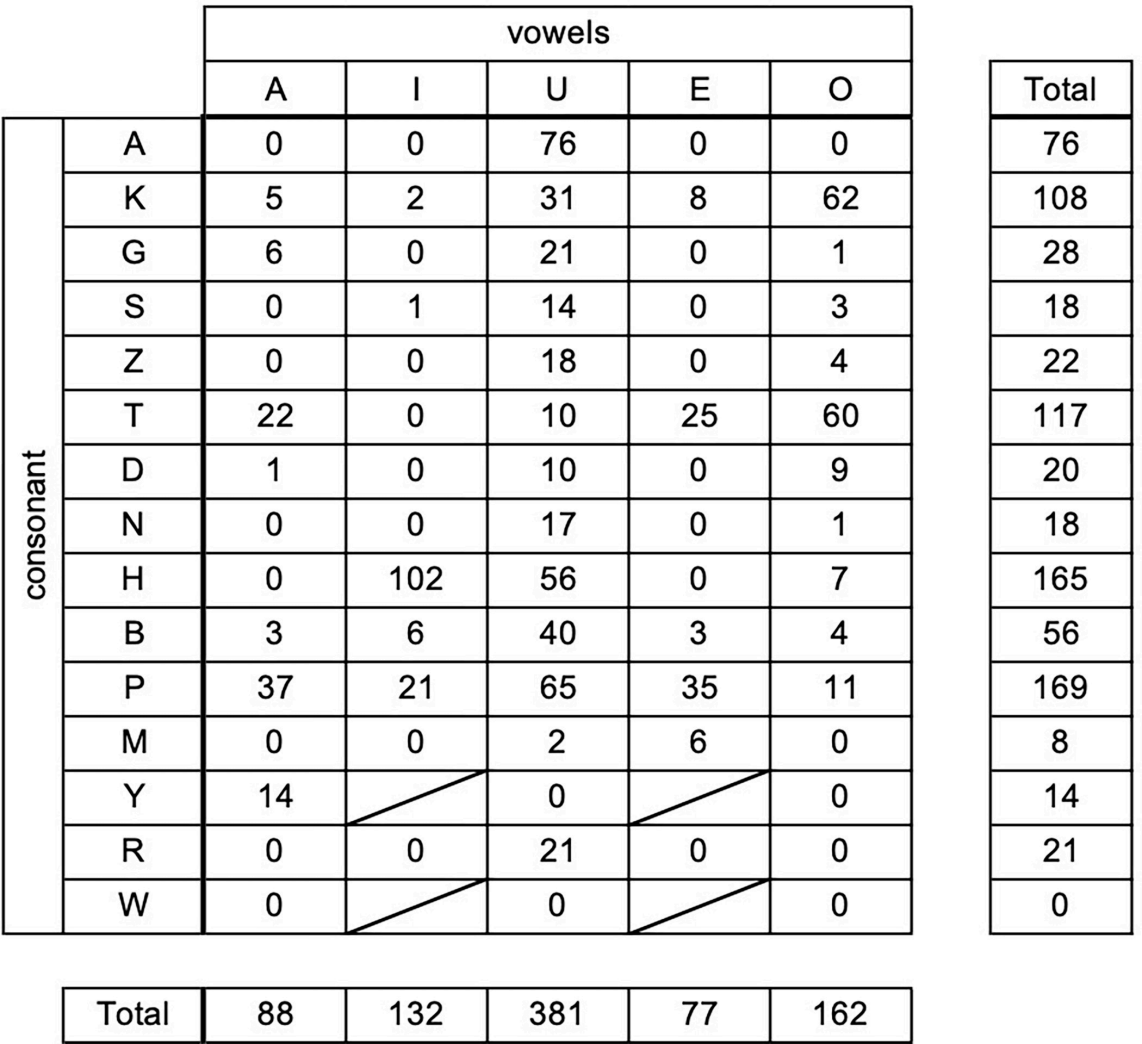
Amount of aerosol particles produced when every Japanese character is read out loud. The total number of aerosols measured by the particle counter at the mouths of three adults (alto, tenor, and bass). A large number of aerosol particles was observed when sounding the vowel /u/, and column “k”, “t”, “h”, and “p” lines.

Next, we analyzed the production of droplet particles produced by vocalization of the Japanese syllabary. For this observation, we selected three study participants (soprano, tenor, and bass) who belonged to an adult chorus group expected to produce more droplets than the other participants. Alto did not participate due to the prevention of the singer’s fatigue and the time required for measurement. The total aerosol particles produced by these three participants is shown in Fig 6. The number of aerosol particles was highest when HI (102) was pronounced, followed by U (76), PU (65), KO (62), and TO (60). When classified by vowel, the numbers of aerosol particles were /a/ (88 particles), /i/ (132), /u/ (381), /e/ (77), and /o/ (162). Regarding the consonants, the numbers of aerosol particles were found most in column “k” (108), “t” (117), “h” (165), and “p” (169). A multivariate analysis was conducted to evaluate the differences in droplet volume between sounding vowels and consonants using all 5 vowels and 14 consonants as variables. The number of aerosol particiles in sounding the vowel line /u/ was 6.70 (95% CI 6.09–32.98) times as much as that produced in sounding the vowel line /a/. No significant differences were observed for the other vowels and consonants.

## Discussion

In our study, the amount and distance of droplets observed during Japanese singing were compared by gender and voice type, as well as by singing in German and calling the Japanese A vowel. Although the flight distance of droplet particles did not differ between the sexes, the number of aerosol particles during singing was significantly higher in female participants than in male. In our observations, female participants produced more ≥ 3 μm and ≥ 5 μm aerosol particles at 1 m in front of the singer than males. Although men have higher respiratory function parameters than women [21], women have a smaller airway cross-sectional area [22]. This smaller cross-sectional area has been shown in computed tomography studies to have a difference in the size of glottal folds. Nevertheless, computed tomography studies have shown gender differences in the size of glottal folds [23], reporting that glottal folds movement was correlated with fundamental frequency in healthy subjects [24]. These results propose that the height of the voice and aerosol production in the glottis are involved in the dynamics of small aerosols. Furthermore, it has been reported that singing at higher pitches produces more aerosols than at lower pitches [25, 26].

The dispersion of droplet particles was observed in front of the singer and 80 cm to the side. In our observation of 20 amateur choristers, while the forward median distance recorded when singing in Japanese was 43 cm, the maximum distance was 61 cm. Additionally, the distance of the droplets was longer but not significant due to the small sample size when singing in German than in Japanese when comparing singers with the same voice types. Therefore, considering that no droplets larger than 5 μm were observed within 1 m in front of the singer in our study, sufficient distance should be ensured when singing in choral groups to ensure safety. This finding is consistent with a meta-analysis showing that a social distance of 1 m or more reduces the risk of SARS-CoV-2 infections [27].

As should be noted from our visualization experiment, the droplets were scattered toward the front and sides. Other studies have also reported that aerosol spread toward the sides during singing [28]. Similarly, we observed that these droplets spread mostly toward the center-right in all three study participants, which may be due to the influence of the dominant arm. However, further studies will need to discuss this issue with more experiments. Our results also suggested that observing droplet dispersion in the horizontal plane and droplet distance to the front was necessary. Hence, based on these findings, the Japan Chorus Association has proposed a stage placement of 2 m in front and 1 m to the side for choral groups singing in Japanese as a distancing guide [29]. Accordingly, Echternach et al. [18] observed aerosol (estimated 0.25–0.45 μm in diameter) dispersion up to 1.4 m in front in 6 seconds after German singers sang “Ode to Joy” and suggested a distance of 2.5 m in front and 1.5 m at the side as safety distance. Furthermore, our study noted that the expansion of trajectory flying droplets was observed in the lateral direction, not floating microlevel droplets. It is important to note that droplet particles ≥ 5 μm have been considered the main cause of droplet infection as well as microscopic droplets at the micro level contribute to the spread of COVID-19 [30]. Thus, it is necessary to address aerosol, conventionally recognized as droplet nuclei, and droplet transmission as infection control measures [14, 31]. During normal speeches, a study also reported that fluid-film bursts in the bronchi produce fine particles, even if the voices were small, further confirming the need for caution [13].

Based on language differences, Alsvd et al. [25] conducted vocalization and singing experiments with 12 Swedish subjects, ranging from opera singers to amateurs, and reported that consonant-enhanced singing produced 1.5 times more aerosol particles than normal singing. The Japanese language lacks consonants pronounced with the labial and lingual teeth, which are used more frequently in English, and is said to have fewer friction sounds [32]. However, research on German singing has shown that the pronunciation of double consonants was longer and stronger in German than in Japanese [33]. Therefore, we believe these factors may explain why the distance and volume of aerosol produced were lower when singing in Japanese. Tanabe also mentioned the strong pronunciation of the “h” consonant in Japanese. In Japanese speech, “k,” “t,” “h,” and “p” produce many aerosol particles. Moreover, the 1–5-μm large particles that make up the bulk of the aerosol originate in the lips, consistent with the fact that more particles were identified in the “p” and “t” column pronunciations [14]. Specifically, the “h” in Japanese is a glottal friction sound, and the number of aerosol particiles produced during articulation may be larger than in other languages. Furthermore, the Japanese /u/ vowel is different from the /u/ vowel in Western languages [34]. Previous reports indicate that the vowel /i/ produces more splashes than /a/ and /u/ [35]. This result may be a difference in pronunciation among Japanese speakers. Notwithstanding, it is likely that /a/ is an open sound and produces fewer aerosol particles in favor of /a/. Moreover, the fact that “t” has more droplets when pronounced and “m” less is similar to the previous report is considered to be because “m” has a lesser aerosol jet. In Japanese singing, fewer aerosol particles were produced in singing the /a/ vowel. Hence, when singing in Japanese in a poorly ventilated environment or in close proximity within a chorus, singing with /a/ vowels and avoiding singing with /u/ vowels may be a solution.

Other visualization experiments have shown that micron-level aerosol generated by coughing can remain airborne for up to 5 min [36]. In COVID-19, fine aerosol produced during eating and conversations are suspended and transmitted indoors [37]. Similarly, although a confined space without ventilation is considered to pose a high risk of infection in chorus singing, it is suggested to control the location of practice and number of participants [38]. However, not all choral rehearsals and stage performances venues have adequate ventilation or space. In addition to regular singing practice, choral practices include vowel singing and lyric reading. Our observational study found that reading aloud had the same droplet flight distance as singing. Thus, infection control measures should be the same for reading aloud and singing. Singing the /a/ vowel is one preventive measure in circumstances wherein the recommended social distance could not be implemented. Notably, wearing a mask strongly suppresses the spread of droplets containing SARS-CoV-2 [39, 40]. We also examined the use of masks during the singing and found no droplets. Therefore, as of December 2021, the Japanese Ministry of Education, Culture, Sports, Science, and Technology recommended using nonwoven masks during choral rehearsals in schools [41]. However, the disadvantages caused by the difficulty in perfecting the music and seeing facial expressions should be understood. Furthermore, considering the effectiveness of vaccines in preventing infection, proactive vaccination is another measure of ensuring safe choral activities [42].

This study has several limitations. First, large individual differences were observed in the distance of droplets and occurrence of aerosol particiles, and the results cannot be applied to each chorus participant. The sample size was small, especially on the observation of horizontal dispersion and singing or speaking in different ways, possibly leading to biases based on individual differences. Moreover, the participants in this study were all Japanese speakers, and this is not a direct comparison with German singing by German speakers. It has been noted that fine aerosols, usually known as droplet nuclei, which are dry and suspended for long periods, are the primary route of the spread of infection in the indoor environment, rather than direct inhalation of droplets. Furthermore, although our study did not observe the dynamics of hovering aerosols in the air for long periods, we provided data on social distance in choral activities. Mechanical ventilation also reduces the amounts of floating aerosols [5]. Therefore, when considering COVID-19 countermeasures, this study can be applied to other languages in the future, and our results should be used as a reference. Nevertheless, individual consideration should be given to the practice venue, hall, and chorus members.

## Supporting information

S1 Fig Measurement using Type-S, visualizing the droplet particles of ≥ 0.5 μm, passing through an area of 20 × 5 cm, in 10 cm and 1 meter in front of the subject’s mouth. The droplets were counted every 1/30 s and recorded every 0.5 s.
(TIFF)

S2 Fig Droplet counts by articulating the Japanese syllabary. Type-S allows the visualization of the droplet, which was set on the mouth of the participants. Observed droplet counts (each 0.2 s) were also shown in the upper mouth, and the sound pressure recorded simultaneously was shown in the lower.
(TIFF)

S3 Fig Visualization of the droplet by singing in German wearing a mask. No droplet from the singer was observed. A few particles floating from the environment were captured in the image after 50 seconds of singing.
(TIFF)
