# Supplementary figures and images for: Expansion of droplets during speaking and singing in Japanese
Source: PLoS One. 2022 Aug 25;17(8):e0272122. doi: 10.1371/journal.pone.0272122 (PMC9409545; doi:10.1371/journal.pone.0272122)

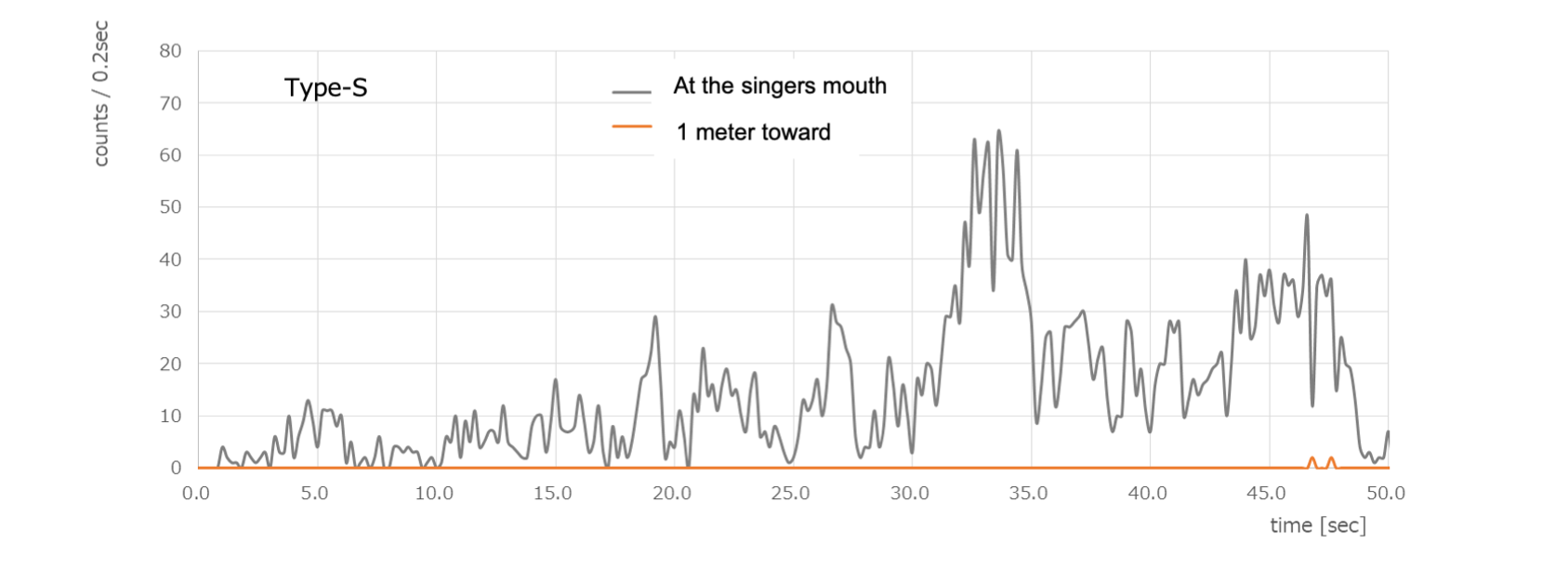

Supplement: S1 Fig — The droplets were counted every 1/30 s and recorded every 0.5 s. (TIFF) [file pone.0272122.s001.tiff]

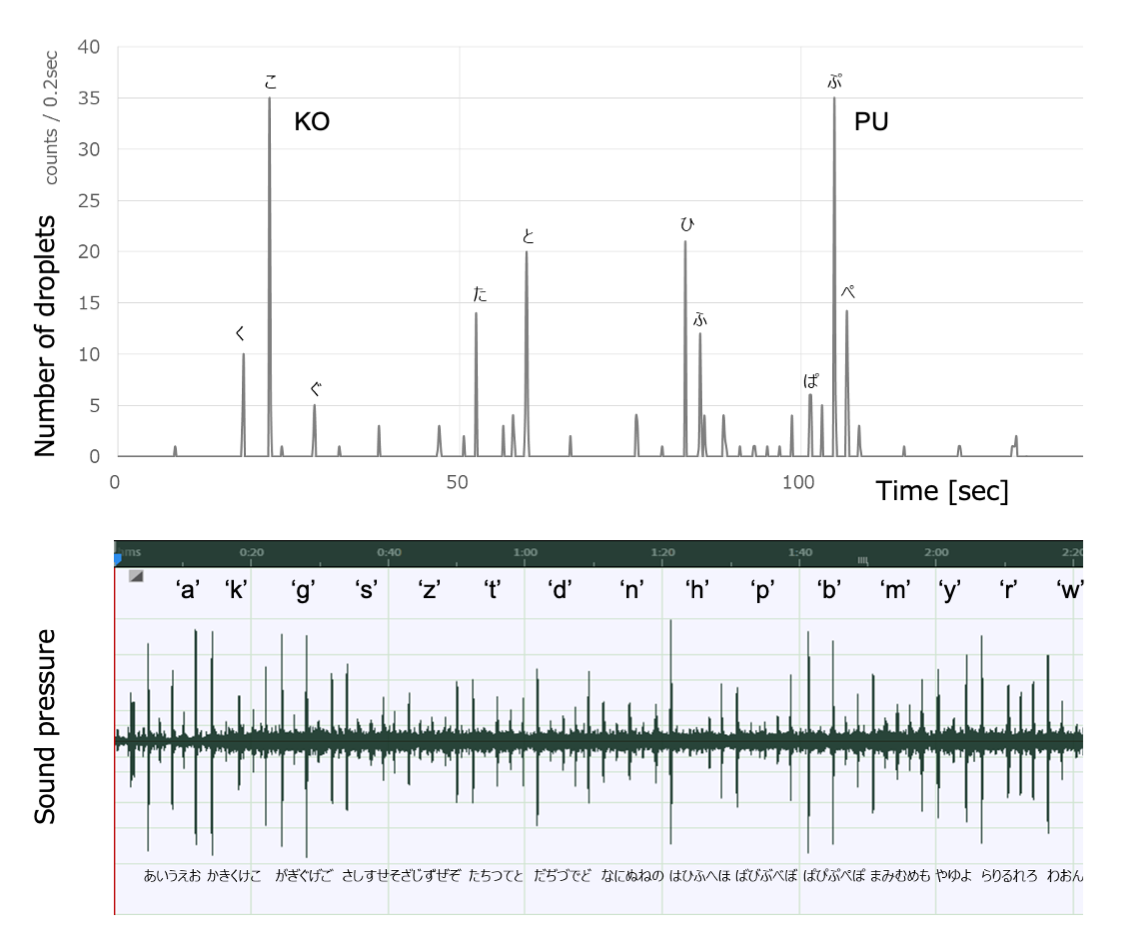

Supplement: S2 Fig — Type-S allows the visualization of the droplet, which was set on the mouth of the participants. Observed droplet counts (each 0.2 s) were also shown in the upper mouth, and the sound pressure recorded simultaneously was shown in the lower. (TIFF) [file pone.0272122.s002.tiff]

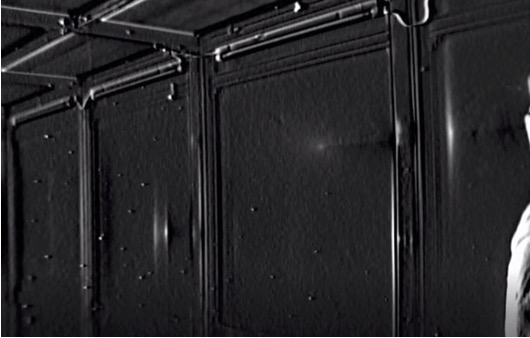

Supplement: S3 Fig — No droplet from the singer was observed. A few particles floating from the environment were captured in the image after 50 seconds of singing. (TIFF) [file pone.0272122.s003.tiff]
